# Overcoming barriers to access and utilization of maternal, newborn and child health services in northern Nigeria: an evaluation of facility health committees

**DOI:** 10.1186/s12913-018-2902-7

**Published:** 2018-02-09

**Authors:** Olugbenga Oguntunde, Isa M. Surajo, Dauda Sulaiman Dauda, Abdulsamad Salihu, Salma Anas-Kolo, Irit Sinai

**Affiliations:** 1 UKaid/Nigeria MNCH2 Programme, No 20 Dawaki Road, Off Ahmadu Bellow Way, Nassarawa GRA, Kano State Nigeria; 2 Palladium, 20 Port Harcourt Crescent, Off Gimbiya Street, Garki, Abuja, Nigeria; 3 Options Consultancy Services Ltd, 2nd Floor, St Magnus House, 3 Lower Thames Street, London, EC3R 6HD UK; 4 Society for Family Health, No 8 Port Harcourt Crescent, Area 11, Garki, Abuja, Nigeria; 5 Palladium, 1331 Pennsylvania Avenue, NW Suite 600, Washington, DC 20004 USA

**Keywords:** Facility health committee, Community structures, Maternal and child health, Northern Nigeria

## Abstract

**Background:**

Poor quality of health services and socio-cultural dynamics may severely limit utilization of health services. Facility health committees were established in several states in northern Nigeria to reduce these barriers. The committees were charged with mobilizing communities, improving quality of health services, and promoting utilization of maternal and child health services. This study assessed this intervention.

**Methods:**

To obtain a comprehensive picture of facility health committees’ influence on maternal and child health services, we selected 33 facilities in three states in northern Nigeria (Jigawa, Kaduna, Kano) where the intervention was active. For each of these facilities we interviewed committee members (*n* = 399), conducted focus group discussions with a subset of committee members (18 focus groups), interviewed facility health providers (two providers from each facility), and conducted client exit interviews (*n* = 501).

**Results:**

Facility health committees appear to have a positive influence on quality of maternal and child health services in the selected facilities. Committee members, health providers, and facility clients all agree that the committees have a tangible positive effect. The most important roles of the committees are to mobilize the community and increase demand for maternal and child health services, in a region where demand is very low. Committee activities further improve health services in many ways, including advocacy, community-facility coordination, fund raising, money donation, and problem mitigation.

**Conclusion:**

Facility health committees can be invaluable in contributing to improved demand for and access to quality maternal and child health services in health facilities in northern Nigeria. They provide strong linkages between community members and the health facilities, directly work to increase demand for services, and address supply-side challenges that often limit utilization of services in health facilities. The intervention can be improved by more broadly communicating committee activities in the community, and by incentivizing facility health committee members.

**Electronic supplementary material:**

The online version of this article (10.1186/s12913-018-2902-7) contains supplementary material, which is available to authorized users.

## Background

Public accountability is increasingly recognized as a pivotal element in improving health system performance globally [1]. In developing countries, where government responsibilities for public services are often not strictly scrutinised, public accountability mechanisms could contribute to improvement in government and health facilities delivering quality health care services [2]. Given the potential benefits of such mechanisms for access and quality of care, governments and non-governmental organizations have invested significant resources in integrating public accountability mechanisms into service delivery, with the aim of improving the overall performance of the health system [3]. Public accountability mechanisms may follow different models, including direct individual involvement, groups, or committees.

Maternal and child health indicators in northern Nigeria continue to be among the poorest in the world. For example, in 2013 infant mortality rate in Nigeria’s North-West region was 89 (per 1000 live births) [4], compared to the African average of 55 and the European average of 10 [5]. Utilization of health services in northern Nigeria is low: only 2.7% of married women of reproductive age in the North-East region reported using a modern contraceptive method in 2013 [4]. These troubling figures persist despite decades of programming designed to increase availability and accessibility of maternal and child health services, improve service quality, and grow the demand for these services.

As part of a large scale five-year UKaid funded health system strengthening programme that ended in 2015, Facility Health Committees were established in three states in northern Nigeria as an accountability platform to improve the quality of maternal and child health services. The implementaion of this intervention was continued and reinforced by the UKaid follow-on Maternal Newborn and Child Health Programme 2. The committees, which are still active, are usually formally constituted with membership that comprises one facility health provider and 12-15 community residents. Members represent all ethnic, religious, age, and gender groups who receive services in the facility. Residents of hard-to-reach locales in the facility catchment area are also included. Members are charged with trying to find solutions to problems that people report about health facilities, as well as with mobilizing the community to improve utilization of maternal and child health services, and sensitizing men and women in the community about the importance of obtaining maternal and child health services in the health facility. Each committee engages with all community groups to understand their views about health services delivery at the facility, decides on a programme of service improvements based on feedback from the community, tells the community what improvements the facility health committee and health facility staff are trying to accomplish, and keeps the community updated on progress toward achieving these improvements. Essentially, these committees are responsible for making it easier for the community to use health facilities and receive quality care.

A literature review of facility health committee initiatives around the world showed evidence that some are more successful than others. They can be highly effective in improving quality of care and health outcomes, but only if they are administered with care. For facility health committees to be successful, clarity of roles, responsibilities, mandates and authority is essential. Systems for accountability must be in place, and facility staff must be sensitized to the committee concept [6]. The full model includes the interplay between health system characteristics, contextual factors, societal attributes and norms, and process elements.

This study retroactively assessed the facility health committee intervention that used a committee approach to increasing public accountability in northern Nigeria. We examined stakeholder perspectives of the facility health committee’s utility in improving the quality of maternal and child health services in three states in northern Nigeria.

## Methods

This study was part of a larger comprehensive mixed methods study that assessed key stakeholders’ perceptions about facility health committees’ contribution to improved quality of health care services, with focus on maternal and child health services. This paper presents mainly the qualitative data from the study. Specific research questions included:

1. What are the specific roles of the committee?
2. What have facility health committees in northern Nigeria accomplished? and
3. Are the facility health committees contributing to improved quality of health care, and if so how? Note that we used a broad definition of quality of care that extends beyond availability and access to care. We defined quality health care as the degree to which availability and access to health care services for individuals and communities increased the likelihood of desired health outcomes.

We considered the perspectives of: (1) facility health committee members, (2) facility health providers, and (3) facility clients.

The study was undertaken in three states in northern Nigeria: Jigawa, Kaduna, and Kano. Jigawa and Kano present very similar health indicators. Like most northern Nigerian states, the population is predominantly Muslim. Socio-demographic characteristics of the population in both states are very similar, and their utilization of maternal and child health services is very low. For example, in 2013, only 7.6% of births in Jigawa and 13.7% in Kano were assisted by a skilled health provider (in the facility or at home), and only 4.6 and 7.8% of children age 12-23 months in Jigawa and Kano respectively were fully immunized [4]. The Kaduna population is more heterogenic, with about half the population following Islam, and the other half belonging to various Christian denominations. Maternal and child health indicators are low but are significantly higher than Jigawa and Kano (35.5% of births with a skilled birth attendant, and 35.3% of children age 12-23 months fully immunized) [4].

Facility health committees were established in all three states between 2010 and 2015. We selected 11 facilities from each state (for a total of 33 facilities) for inclusion in the study as follows:

- One secondary facility, with a facility health committee operating for at least two years, was randomly selected from a list of all such facilities in each state; and
- 10 primary health care facilities, with a facility health committee operating for at least two years, were randomly selected from a list of all such facilities in each state.

Four data components were collected from each of the 33 selected facilities:

1. **Survey of facility health committee members:** We interviewed all current members of the facility health committees in the 33 selected facilities, who are community members (excluding the committee member who is the facility health provider). Since 12-15 community members participate in each committee, we expected a sample of 400-500 respondents. Final sample size was 399 committee members, representing a response rate of about 90%.
2. **Focus group discussions with facility health committee members:** To gain a deeper understanding of facility health committee members’ views and perceptions, we selected a subset of facility health committee members from the 33 facilities to participate in focus group discussions. Again, we excluded those committee members who are health providers to minimize respondent bias. Convenience sampling was used to select participants. Six focus group discussions were conducted in each state, for a total of 18 focus groups discussions. Each focus group consisted of 3-8 committee members. In Kaduna and Kano, four groups consisted of male participants, and two groups consisted of female participants. In Jigawa, the groups were mixed gender, with 2-3 female participants per focus group.
3. **In-depth interviews with health providers**: We interviewed two maternal and child health providers from each of the 33 facilities, for a total of 66 interviewed providers. In cases where more than two eligible providers were working in the facility, two were randomly selected to participate in the study.
4. **Facility client exit interviews:** Female clients were intercepted as they left the facility after receiving services. From each of the 33 facilities, we attempted to interview: five women who came to the facility for antenatal care services; five women who came for family planning; and five women who came for child health care (either immunization or sick child). This approach was not strictly followed because not all services were available every day of the week, and because some women came for more than one purpose or received maternal and child health services, but the primary reason for their visit was different.

Instruments for all study components (Additional files 1, 2, 3 and 4) were designed to elicit information on respondents’ and participants’ perceptions regarding facility health committee roles and responsibilities, the utility of the committees in improving quality of care and facility services, and how the committees could be improved. Again, our broad definition of quality health care included availability and access to services, as well as other elements such as provider knowledge and attitude and characteristics of the facility.

Fieldwork was undertaken in early 2016, beginning with a thorough training of all interviewers, facilitators, and supervisors. Female interviewers interviewed female respondents, and male interviewers interviewed male respondents. All interviews and focus group discussions were undertaken in Hausa, the most commonly used language in the study areas. Focus group discussions and in-depth interviews were audio recorded.

### Analysis

To analyse quantitative data (committee member interview, client exit interview), we used simple frequencies and cross-tabulations. Significance levels were tested using F-tests and χ^2^ as appropriate. To analyse qualitative data, all recordings of focus group discussions and in-depth interviews were transcribed into English. Analysis was iterative, coding the transcripts by thematic content to identify emerging themes.

### Ethical considerations

Prior to commencing fieldwork, all study protocols and instruments were approved by each state’s Health Research and Ethics Committee. While there was no physical risk associated with study participation, all efforts were made to ensure confidentiality of responses. All interviewers and facilitators received training on the intricacies of undertaking research involving human subjects, as part of their overall training. Interviews and focus group discussions were undertaken in private places, where the conversation could not be observed or overheard.

All respondents and participants provided informed consent before their interview or focus group. Focus group participants consented privately rather than in a group context to avoid peer pressure to participate. The consent form explained the purpose of the study and made it clear to participants that they do not need to participate if they do not wish to, that they are free to not respond to questions that make them feel uncomfortable, and that they could stop the interview at any time. It also articulated that the information they provide will be kept confidential and that, while the data they provide would be associated with the specific facility, individual participants’ responses would not be identifiable in the data.

For facility committee members, the consent form also clarified that their decision to participate would not affect their position in the committee. Those facility committee members who participated in the focus group discussions were told that their information will remain as confidential as group participants make it, and that they should not repeat the conversation to people outside the discussion. Interviewed providers were assured that their position in the facility would not be influenced by their decision to participate in the study. Similarly, clients were told that their agreement to participate would not affect services they receive in the facility.

## Results

### Facility health committee members’ perspective

#### Facility health committee composition and operations

Our findings about the perceptions of committee members are based on interviews we conducted with them (quantitative), and from focus group discussions (qualitative). We present the story that emerges from both sets of data together. Table 1 shows the characteristics of committee members who were interviewed (*n* = 399). Since we interviewed almost all committee members from the 33 selected facilities, this profile provides a good representation of facility health committee composition overall. Committee members represent community members from all walks of life, including farmers, traders, artisans, and civil servants; some have received higher education, while some have received no education. Most committee members were Muslim (100% in Jigawa and Kano), reflecting the religious distribution of the population in the three states. Age ranged from 20 to 85 years, with a mean of 43.2; the majority of members (65%) were between the ages of 30 and 50 years.

**Table 1:** Facility health committee members' profile

	Interviews (*n*=399)
Mean age	43.2
% female^a^	19.8
% Muslim	92.5
% with Qur’anic education or no education	34.6
% with secondary or tertiary education	30.3
% farmer	28.8
% trader	22.6
Mean years of committee memberships	4.4

^a^ Gender of respondents was not noted in the questionnaire. This figure is the proportion of females in the facility health committees of the 33 selected facilities. Since response rate was about 90%, this figure approximates the percent of interviewed women.

On average, members have been serving in the committees for 4.4 years. Focus group discussions reveal that committee members are community members who are community service-minded. Most have done volunteer work before, and they joined the committees through a process of nomination and election.

“After meetings and consultations, the community decided to elect me to the facility health committee.” (Kaduna, 35-39)

The facility health committees generally meet monthly to discuss ongoing and emerging problems with health services offered in their facilities. Their concerns are easily communicated to facility staff, since the facility in-charge officer is a member of the committee. Almost all survey respondents (97.5%) said their committee also holds meetings with health facility staff.

#### Facility health committee roles and responsibilities

Committee members are highly motivated and strongly believe in the usefulness of their committee. Almost all (98.5%) of survey respondents affirmed that the establishment of a facility health committee can influence the quality of healthcare services in health facilities, and 89% believed this influence to be significant. Respondents were asked whether facility health committees could contribute to improving quality of care in health facilities through specific pathways. They were also asked about the influence that their own committee had on quality of care, through these same specific pathways, in the year preceding the survey. Their responses, shown in Table 2, suggest that committee members perceived that committees could help improve facility health services through a broad range of activities, and that they had experienced success with such activities in their own committees. These activities include increasing access to and improving the quality of services, as well as demand creation within the community.

**Table 2:** Committee members' perspective: ways that facility health committee can contribute to improving quality of services in health facilities (%)

*n*=399	Facility health committees in general	Experience of respondent’s own committee in past year
Enable community mobilization	72.9	70.9
Increasing health provider availability	60.9	54.1
Improve health provider attitude	60.2	59.4
Help providers do a better job	57.1	57.6
Increase access of community members to services	49.4	49.9
Facilitate renovation of the facility	49.4	43.4
Improve availability of equipment	46.9	40.9
Expand range of services provided	40.1	38.8
Reduce stock outs of medicines or commodities	36.3	35.6
Increase days or hours of operation	33.3	30.6
Facilitate outreach services	33.1	30.3
Facilitate home services by health workers	29.3	28.1

These findings were confirmed in the focus group discussions, where most participants viewed community mobilization and demand creation as the most important roles for the committee.

“The aim of creating this committee is to enlighten the people on health-related issues because our people are rejecting immunisation services.” (Jigawa, 25-29)

“The role of the committee is to help improve the conditions in the hospital by creating awareness in the community.” (Kaduna, 25-29)

However, committee activities overall were very varied, as the following focus group discussion quotes show. They even included raising money from the community to compensate health workers during periods in which the Ministry of Health did not pay them wages, which is a common problem in northern Nigeria.

“We are the ambassadors of the community in the facility. We are encouraging members to respect the health care providers.” (Jigawa, 20-24)

“By taxing ourselves, we [are able to] provide [the] equipment and tools necessary for running of the facility smoothly.” (Jigawa, 60-64)

“We normally use our private vehicles to transport patients to the facility and for referral services.” (Jigawa, 55-59)

“We are also responsible for payment to some staff […] Also we renovated the roofing of this facility.” (Jigawa, 35-39)

“We carry sick people from home to hospital. Sometimes, we do the reverse by inviting our health workers to treat a sick person at home.” (Kaduna, 35-39)

“We also serve as middlemen between the community and health workers, and organise people to clean the hospital environment.” (Kaduna, 35-39)

“We visit prominent people in the community to [advocate for them to] help the facility when the need arises.” (Kaduna, 25-29)

“Regular supervision by the committee members can also help improve quality of services. We also meet with in-charge officers if there is any urgent need.” (Kano, unknown)

“We established a community forum to mobilise well-to-do people within the community, to assist the hospital.” (Kano, unknown, )

**Fig. 1:**
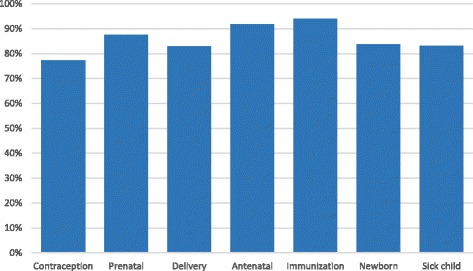
Committee members' perspective: facility health services that can be improved through facility health committee work

Survey responses indicate that committee members believed that facility health committees helped improve services across all maternal and child health services, as shown in Fig. 1.

#### Facility health committee interaction with facility staff and the community

Overall, committee members appear to have a good relationship with facility staff. The majority (88%) of survey respondents said that facility staff have been very receptive to recommendations made by the facility health committee (8% said somewhat receptive, and fewer than 2% said not receptive). Similarly, 92% of respondents said that if the facility health committee made a recommendation aimed at improving service quality, the facility staff would undertake immediate measures to implement the recommendation.

Focus group discussions provided a more complex picture. It appears that, in some cases, initial health provider attitudes were negative, but these improved with the dedication and perseverance of committee members, who were trained (as part of establishing the committee), on how to address such issues as soon as they emerge.

“Initially we had problems with [the health providers] regarding dedication and negligence to duty. We raised our concern and resolved this once and for all.” (Jigawa, several)

“We initially encountered hitches with [the health providers] in terms of running 24-hour services, which we later resolved amicably” (Jigawa, 35-39)

In their efforts to educate the community about available facility services and the importance of facility delivery, prenatal care, and immunization, committee members sometimes faced opposition. Again, through dedication and perseverance they continued to create a gradual shift in community perceptions about the importance of utilising available services at the health facility.

“Initially we faced challenges of cooperation with community members because they thought that we were being paid for the services. But we kept on convincing them.” (Jigawa, 35-39)

Overall, however, facility health committee members reported significant support from the community.

“We are receiving enough support from the community. When anything is beyond our capabilities, [the community] helps accordingly.” (Jigawa, several)

“The community is contributing a lot to improve the services rendered in our hospital. They helped in building a ward and paying other utility bills like electricity and water bills.” (Kaduna, 25-29)

#### Facility health committee successes and challenges

In focus groups discussions, we asked participants about their perception of the successes of their committees, and the challenges they faced. As the following quotes show, committee members believed that people in the community trusted them. They also noted that the composition of membership of the committee, which ensured adequate representation of all ethnic groups in their catchment areas, was helpful in bringing different segments of the community together to achieve the desired goals.

“The inclusion of relevant ethnic groups to the facility health committee is very encouraging to all of us. Each one is playing his role to yield better result.” (Kaduna, 35-39)

As for challenges, committee members listed few. While health providers and community members initially did not trust them, they said this changed following positive outcomes of committee interventions. A persistent challenge, however, is that the cost of repairing or replacing equipment could be somewhat prohibitive. As a result, some committee members felt they were not accomplishing as much as they would like.

“We failed to provide laboratory services and blood transfusion services, because the equipment were very expensive and we couldn’t afford buying them. We tried to reach the Senator representing […] to help us, but all efforts failed.” (Jigawa, 50-54)

These findings are echoed in survey results, where the most frequently cited reasons for success of the facility health committees were support of the community (89.3%), commitment of committee members (77.6%), and support of facility staff (74.7%). The most frequently cited reason for failure in some committee activities were inadequate funding (61.2%) and insufficient training (28.1%). All other listed determinants of success or failure were mentioned by fewer than 20% of respondents.

### The service providers' perspective

#### Facility health committee roles and responsibilities

In-depth interviews with providers confirmed the important role that facility health committees play in improving facility maternal and child health services. Committee functions listed by providers include: (1) create awareness and mobilize the community, (2) improve linkages between the facility and the community, (3) improve the supply of commodities and consumables, and (4) facilitate maintenance of infrastructure and equipment.

“In my opinion, [the role of the facility health committee] is calling to the attention of community members issues regarding immunization, and maternal issues such as antenatal visits and delivery at the facility. Most of the time, their wives deliver at home. There was a case that I referred to [the health facility] but they refused to go and decided to deliver at home with the assistance of a patent medicine vendor. This is not good. The committee should continue with mobilising the community.” (Health care worker, Jigawa)

“The role of this committee is to serve as a link between hospital and community.” (Community health worker, Kano)

“Their role is in the provision of water and electricity to the facility. Currently they are working toward the provision of an ambulance for the facility. They are also working toward the supply of electricity to this facility, because we have two generators in this facility but we can’t sustain fuelling them.” (Health care worker, Jigawa)

#### Facility health committee successes and challenges

When asked for examples of specific cases of facility health committee success, the interviewed health care providers mentioned a long list of examples. These examples all fall under the four categories mentioned above. One provider also said committee members serve as role models by practicing what they preach: they and their families use facility health services, and in doing so are an example to everyone else.

Consistent with committee members’ views, insufficient funds for activities is an important concern for providers. When asked what can be done so that the facility health committees further improve quality of facility health services, providers mentioned making available additional funds. Providers also suggested that committee members be compensated, with some even suggesting that committee members be employed.

“You know that this work they are doing is voluntary. Most of them complain of not being given incentives. Not everybody is committed. If they are given incentives, they will put more effort in their work.” (Health care worker, Kaduna)

#### Facility health committee and provider interaction

Most health providers interviewed reported hearing about committee recommendations through the facility in-charge officer, who is a member of the committee. Most were happy with this arrangement and expressed appreciation for facility health committee’s work. However, some suggested that the committee did not meet regularly, and others recommended that they directly interface and communicate with health providers, rather than channelling this information through the facility’s in-charge officer.

Most facility service providers reported a cordial and respectful relationship with committee members. While holding service providers accountable for providing good services is an important role of the committees, this is not reflected in provider responses, which is not surprising.

“Yes, we all respect each other. They visit us from time to time and listen to our problems. Our relationship with them is cordial. Facility health committee members really respect the health providers in the facility. They do respect us and we work as a team.” (Facility health worker, Kano)

When asked what could be done to further improve facility health committees, many respondents mentioned incentivizing committee members. Other improvements suggested by providers include further (unspecified) training of committee members and more involvement from the government.

“I want the government and NGOs to invite them to attend some workshops and training, to make them to know their roles, so that they can be more effective.” (Health care worker, Kaduna)

“…provision of incentives to the members and communities can also improve the services.” (Health care worker, Kano)

### The facility clients' perspective

We interviewed a total of 501 women as they left the facility after receiving services. Their median age was 25 years (range: 15-71), and their median number of births was 3 (range: 0-17). Most (87.0%) were Muslim (100% Muslim in Jigawa and Kano), and about one quarter had never attended school.

The most commonly cited reasons for their visits on the day of the interview include antenatal care (34.7%), family planning (21.2%), immunization (17.8%) and having a sick child (17.6%). Other reasons cited were being sick, follow up, and postnatal care.

#### Clients' satisfaction with services

Clients were asked about their satisfaction with various aspects of facility services. The level of satisfaction (i.e., “highly satisfied” or “satisfied”) for each item is shown in Table 3. Almost all clients were satisfied with most aspects of the services they received in the facility. We created a satisfaction scale that combined responses to all of the items. To do so, we added 2 when “highly satisfied” and 1 when “satisfied” for each item, and divided the total by 28 (as there are 14 factors included, and each can be coded 0, 1 or 2), to arrive at a scale ranging 0-1, with a mean of 0.66, and a Cronbach Alpha value of 0.855. Comparing the mean score for each state, we see the highest score (0.74) for Jigawa, followed by Kano (0.66), and Kaduna (0.57); these differences are statistically significant at the *p* < 0.01 level.

**Table 3:** Clients' satisfaction with health facility service areas

*n*=501	Satisfied or very satisfied (%)
Condition of waiting area	96.8
Attitude of provider	96.6
Visual privacy of examination room	96.6
Provider response to health concern	96.2
Time spent with provider	95.0
Waiting time	94.4
Audio privacy of consultation	94.2
Opening and closing hours of facility	93.0
Cost of services or treatment	88.4
Availability and condition of lavatories	87.5
Availability of medicine	86.4
Condition of examination rooms	83.4
Availability and condition of hand washing facilities	55.9

#### Clients' perceptions of the committee

A third of respondents (33.4%) were aware that the facility had a facility health committee. Again, there were statistically significant differences between the three states. In Kaduna, 46.7% of respondents were aware that the committee existed, compared to 28.2 and 25.7% in Jigawa and Kano respectively (χ^2^ = 18.699; p < 0.01). The 169 respondents who answered in the affirmative were asked where they heard about the committee. The most frequently cited sources of information were friend, neighbour, or relative (39.2%), health provider (33.1%), and community activity organized by the committee (17.5%).

Respondents who had heard of facility health committees were also asked about the influence of the committees. Almost three-quarters (73.1%) said that the committee significantly influences the way health services are delivered in the facility. Specific service provision aspects and health services that they thought were directly improved as a result of committees’ interventions are shown in Table 4. Clearly, they saw significant improvements in many aspects of the service in most service areas, with the exception of caesarean sections.

**Table 4:** Clients' perception of service areas and health services that improved as a result of facility health committee work

*n*=169	Perceived improvement (%)
Service provision aspects:	
Helped providers do a better job	74.3
Enabled community mobilization	60.4
Increased health provider availability	58.3
Increased access of community members to services	57.6
Improved health provider attitudes	53.5
Reduced stock outs of medicines/commodities	45.8
Facilitated outreach services	43.1
Expended range of services provided	38.5
Increased days or hours of operation	37.5
Facilitated renovation of the facility	37.5
Facilitated home services by health workers	37.5
Improved availability of equipment	35.4
Health service:	
Prenatal services	100
Sick child care	100
Immunisation services	98.2
Newborn services	98.2
Delivery of babies	95.2
Antenatal services	90.4
Contraceptive provision	86.2
C-sections	18.0

## Discussion

Our findings present a consistent picture of an intervention that successfully contributes to improving infrastructure and quality of care. While we do not have service statistics data, respondents’ perceptions suggest that the intervention also contributes to increased demand for maternal and child health services in northern Nigeria. Facility health committee members, facility health providers, and facility clients all agree that the committees are helpful in improving many aspects of care for a broad range of services. This is consistent with findings from other studies, where facility health committees were found to contribute to improved service quality and access to care [2, 3, 6–8]. The diversity of committee members in our study, who represent the spectrum of facility clients, as well as committee formation through nomination and election, also reflect findings similar to other studies.

While some studies of facility health committees in various countries described poor or absent linkages between facility health committees and the communities, and low recognition leading to poor performance [9–13], our study found that, despite the fact that only a third of community members know about their existence, facility health committees are successful in providing this linkage. Members are highly motivated, generally have good relationship with providers and with the community, and their work is respected and appreciated—all factors previously identified by other studies [6, 7] as contributory to the success of facility health committees. The fact that community members in our study communities participated in electing the facility health committee members may have also contributed to the support and harmonious working relationship between committee members and the community, resulting in the measure of successes recorded. This is consistent with the finding of Zakus and Lysac (1998) who noted that the processes of selection of organization members, representativeness and the degree to which they represent local issues are critical to community members’ perception of their legitimacy [14], which may influence offers of support to committee members.

As similarly reported elsewhere [7, 15], despite the generally good relationship between committee members and service providers in our study, there were a few instances of tension, especially during initiation of facility health committee activities. Committee members described instances of negative attitudes among service providers toward their recommendations on certain areas of service delivery, such as facility opening hours and staff dedication to duty. The successful management of these challenges was attributed to members’ training and their resilience. This emphasises the need for adequate and continuous capacity building for health facility committee members to enable them to deliver on their mandates [3, 15, 16].

Across the board, funding appears to be the most significant challenge for facility health committees. From the providers’ perspectives, funds are needed to incentivize facility health committee members. Facility health committee members do not mention incentives, but they complain that there is no budget to undertake the necessary infrastructure improvements or to buy needed equipment. In contrast, in a study in Kenya, facility health committee members recommended either payment or increases in allowances, as they perceived this would improve members’ commitment and committee effectiveness [3, 15]. However, a study exploring the roles of facility health committees as a social accountability platform in West and Central Africa reported no difference between committees whose members received remunerations and those who did not [7]. In our study, committee members actively engage in fundraising and often pay for equipment out of their own pockets. While they cannot accomplish all that they set out to do due insufficient funds, they are nevertheless still able to accomplish a lot.

Only a third of clients in our study have heard of the existence of the facility health committee. Other studies [2, 3, 15, 17] have reported similar low levels of awareness of facility health committees among facility users and a likelihood of higher proportion among the larger community members served by those committees. This could result in communication gap and lack of information sharing between committee members and the community, thus limiting the functionality of the committees. The Kenyan study noted that women and relatively less well-educated respondents were less likely to be aware of the facility committees [3]. In our study, low awareness of facility health committees may also be related to respondents’ low level of education, as only a quarter of respondents had ever attended school. This assertion is confirmed by the relatively higher awareness level in Kaduna, where women are more educated. For the committee to be most effective, clients need to know about their existence and be able to approach committee members with any problems that they encounter in the facility or concerns they have about the care they receive. This suggests a need for awareness creation about facility health committees within the community, which would improve communication and the potential for committees’ success in improving quality of care.

Client satisfaction with maternal and child health services is very high in our study, almost too high to be believable, especially given the actual physical conditions and the state of service delivery in these facilities. Yet these results are consistent. Caesarean section is the one service that facility health committees cannot do much to improve, and it is also the one service for which clients did not perceive improvements. While we interpret these results with caution, they lead us to believe that client satisfaction is indeed high. We posit that clients have low expectations for service quality and are happy with any service that meets or exceeds their low bar. If this is the case, then it may also explain the differences between states in client satisfaction with services. Kaduna’s population is more heterogeneous and better educated than that of Jigawa and Kano. It is not surprising, therefore, that clients in Kaduna have higher expectations for quality of care and are therefore less satisfied with current services. On the other hand, their lower satisfaction may also be explained by the higher proportion of Kaduna clients who had heard of facility health committees. Clients who heard about facility health committees may be more likely to understand that improving conditions is within the committee’s capabilities, and their expectations for quality care may increase.

An important limitation of the study is data availability. Results would have been stronger if we compared client satisfaction with maternal and child health services with clients of facilities in control areas, where no facility health committees were established. Also, if we knew the date in which each committee was established, we would have been able to compare service statistics for key indicators before and after the committees were created in order to evaluate changes in uptake of services. However, the committees were created at various times during a five-year period. We do not have the specific dates, and it would be difficult to get access to historical data from before and after facility establishment. Therefore, we are unable to examine service utilization figures.

Despite these limitations, we are encouraged by our positive results. Facility health committees in northern Nigeria are clearly effective in improving maternal and child health, as recognized not only by the facility members themselves, but also by facility health providers and facility clients. We posit that the facility health committee intervention can be sustainable. After initial establishment of committees and mentoring of members, committees can continue with minimal support, if any, because committee members are so dedicated and empowered, and because they manage to raise funds for equipment and services that otherwise would remain unfunded.

## Conclusions

Several programmatic implications flow from our findings. The facility health committee model used in northern Nigeria appears to be effective in improving maternal and child health service quality of care, creating awareness and motivating community members to improve utilization of services at health facilities. It should be scaled up to more facilities and additional states for broader impact. However, it can be improved by communicating to community members the facility health committee’s presence, responsibilities, and authority. This will make committee members more accessible to the community, who in turn will be able to convey any issues they have at the facility more easily, enabling committee members to take steps to rectify these issues.

The facility health committees will also benefit from increased funding. While committee members are very dedicated, facility health providers strongly believe that they will be even more motivated if incentivized. If funding is not available to compensate them for their time, they should at least be given recognition for their volunteer work. For example, they can be given certificates of appreciation from the community, or could be recognized in community meetings. While health providers suggested that facility health committee members could become employees, we do not recommend this approach, as it will take away their advantage of not being part of the government establishment, which allows them to better ensure accountability.

## Additional files

Additional file 1: Survey Questionnaire: Client Exit Interview. (DOCX 49 kb)

Additional file 2: Focus Group Discussion Guide with FHC members. (DOCX 22 kb)

Additional file 3: In-depth Interview Guide with health providers. (DOCX 29 kb)

Additional file 4: FHC members survey questionnaire. (DOCX 107 kb)
