# Supplementary material for: Overcoming barriers to access and utilization of maternal, newborn and child health services in northern Nigeria: an evaluation of facility health committees
Source: BMC Health Serv Res. 2018 Feb 9;18:104. doi: 10.1186/s12913-018-2902-7 (PMC5807838; doi:10.1186/s12913-018-2902-7)
Supplement: Supplementary file 1 — Survey Questionnaire: Client Exit Interview. (DOCX 49 kb) [file 12913_2018_2902_MOESM1_ESM.docx]

**MCNH2**

**Facility Health Committee Study**

**Patient exit interview**

Provider code

Provider gender (circle one) Male / Female

Interviewer code

Name of facility ___________________

Today’s date _______ / _______ / _______

Day Month Year

Let’s start with some questions about you

| **No.** | **Questions and filters** | **Coding categories** |
| --- | --- | --- |
| 1 | How old were you on your last birthday? | Age |
| 2 | How many years have you attended school? | Years at school  Never attended school 0 |
| 3 | What is your main occupation, that is, what kind of work do you mainly do? | Agriculture 1  Labor/industry/technical 2  Sales (street, market) 3  Sales (shop) 4  Services 5  Professional/administrative 6  Other ___________________________ 9  (specify) |
| 4 | What is your religion? | Muslim 1  Catholic 2  Other Christian 3  Animist 4  Other _______________________________ 9  (specify) |
| 5 | How many children have you given birth to (have your wives given birth to) who live with you? |  |

The next set of questions is about the services in this facility

| 6 | What brought you to the facility today? | I am sick 1  My child is sick 2  Follow up from a previous sick visit 3  Pregnancy test 4  Prenatal care 5  Antenatal care 6  Immunization 7  Other _______________________________ 9  (specify) | | | | | |
| --- | --- | --- | --- | --- | --- | --- | --- |
| 7 | How long have you been coming to this facility for health services? | Less than 1 year 1  1-3 years 2  3-5 years 3  More than 5 years 4 | | | | | |
| 8 | In general (most of the time), how satisfied are you with the following aspects of services offered in this facility?   1. How long you wait to see the provider 2. The condition the waiting area 3. Ability to discuss your health concern with the provider 4. Provider’s response to your health concerns 5. The amount of time you can spend with the provider 6. Explanations you receive from providers about your health concerns 7. Privacy from having others see your examinations 8. Privacy from having others hear your consultation discussions 9. Availability of medicines in the facility 10. The number of days in the week that the facility is open 11. The hours that the facility is open, that is, when they open and close 12. The condition of the examination rooms 13. The availability and condition of lavatories in the facility 14. The availability and condition of places where you can wash your hands in the facility 15. The availability of medicines in the facility 16. Cost for services or treatment | Very satisfied | | Satisfied | Unsatisfied | | Very unsatisfied |
|  |  | 1  1  1  1  1  1  1  1  1  1  1  1  1  1  1  1 | | 2  2  2  2  2  2  2  2  2  2  2  2  2  2  2  2 | 3  3  3  3  3  3  3  3  3  3  3  3  3  3  3  3 | | 4  4  4  4  4  4  4  4  4  4  4  4  4  4  4  4 |
| 9 | This facility has an FHC. Did you know that this facility has an FHC? | Yes 1  No (skip to Q11) 2 | | | | | |
| 10 | How did you hear that the facility has an FHC? | Provider told me 1  A friend/neighbor/relative told me 2  There was a notice in the facility 3  The FHC organized a communty activity 4  Everyone knows there is an FHC 5  I noticed changes in the facility 6  Other _______________________________ 9  (specify) | | | | | |
| 11 | HFCs are created in order to improve the way services are organised and offered in health facilities. In your opinion, how much influence can FHCs have on health serve quality? | Significant influence 1  Limited influence 2  No influence 3 | | | | | |
| 12 6 | What are ways that FHCs can contribute to services? (circle all that apply) | Increase health provider availability A  Help providers do a better job B  Improve health provider attitudes C  Expend range of services provided D  Increase days or hours of operation E  Increase access of community members to services F  Reduce stock outs of medicines/commodities G  Improve availability of equipment H  Facilitate renovation of the facility I  Enable community mobilization J  Facilitate home services by health workers K  Facilitate outreach services L  Other _______________________________ Z  (specify) | | | | | |
| 13 | Do you think your HFC influences the way health services are delivered in the facility, or by facility providers in the community? | Significant influence 1  Limited influence 2  No influence 3 | | | | | |
| 14 | Have you noticed any specific changes in the way health services are delivered in the facility in recent years? | Yes 1  No (skip to Q18) 2 | | | | | |
| 15 | Do you think these changes could be the results of the FHC’s recommendations? | Yes 1  No 2  I don’t know 8 | | | | | |
| 16 | In what ways do you think your FHC activities have influenced the delivery of services in the facility? (circle all that apply) | Increase health provider availability A  Help providers do a better job B  Improve health provider attitudes C  Expend range of services provided D  Increase days or hours of operation E  Increase access of community members to services F  Reduce stock outs of medicines/commodities G  Improve availability of equipment H  Facilitate renovation of the facility I  Enable community mobilization J  Facilitate home services by health workers K  Facilitate outreach services L  Other _______________________________ Z  (specify) | | | | | |
| 17 | Think about specific service areas in the facility. Have the FHC improved service quality in the following service areas:  (a) Contraceptive provision  (b) Prenatal services  (c) Delivery of babies  (d) C-sections  (e) Antenatal services  (f) Immunization services  (g) New-born care  (h) Sick child care | Yes | | | | No | |
|  |  | 1  1  1  1  1  1  1  1 | | | | 2  2  2  2  2  2  2  2 | |
| 18 | Overall, do you think the committee has been successful in carrying out its responsibilities in improving quality of service delivery at the facility? | Yes 1  No 2 | | | | | |
|  | | | | | | | |
|  | | |  | | | | |
