# Supplementary material for: Overcoming barriers to access and utilization of maternal, newborn and child health services in northern Nigeria: an evaluation of facility health committees
Source: BMC Health Serv Res. 2018 Feb 9;18:104. doi: 10.1186/s12913-018-2902-7 (PMC5807838; doi:10.1186/s12913-018-2902-7)
Supplement: Supplementary file 2 — Focus Group Discussion Guide with FHC members. (DOCX 22 kb) [file 12913_2018_2902_MOESM2_ESM.docx]

**MCNH2**

**Facility Health Committee Study**

**Member focus groups**

State: __________________

Focus group #: ___________

Number of participants _____

Number who are female ____

*You are all members of a FHC in this state, and all of you participated in our survey recently. We would like to talk to you some more about your experiences as an FHC member, your perceptions about FHC accomplishments, and how FHCs can be improved. This will give us more in-depth understanding about these issues than we could get from the survey alone. We selected for these focus groups FHC members with a range of views about FHCs, so we should have an interesting discussion.*

WARM UP

1. How long have you been a member of a FHC?
2. How did you decide to get involved in the FHC?
3. What are your other interests?

FHC MANDATE AND AUTHORITY

1. What do you think is the role of FHCs? What are they meant to accomplish?
2. How much can FHCs influence quality of services?
3. What are the ways the FHCs can help improve quality of services? How else can they contribute to services? What else can they do?
4. How does your HFC engage with the facility when it has a recommendation to improve care? How can the process become more effective? How can it be streamlined?

INDIVIDUAL CONTRIBTION TO FHC

1. What is the contribution of individual members to the FHC mission?
2. Is the number of members in the FHC about right? Would the FHC be more productive if it had fewer or more members?
3. Are all the relevant ethnic, demographic, economic, and other population groups in the catchment area of the facility represented in the FHC?
4. Do you feel that FHC members get enough support from the community?
5. Do you feel that FHC members get enough support from facility staff?

SUCCESS AND FAILURE OF FHC

1. How successful was your committee in improving services in the health facility?
2. Please tell me about specific instances where your FHC was successful in improving services in the facility? (probe re maternal and child health, if this does not come up spontaneously)
   1. What services were improved?
   2. How where they improved?
   3. How exactly did the FHC affect the improvement? What was the process that the FHC undertook to arrive at specific recommendations, and to influence the facility that the change is beneficial?
   4. How did individual FHC members contribute to the process?
   5. Where there any challenges in the process? What were the challenges? How were they overcome?
3. Please tell me about specific instances where your FHC tried to affect change but was not successful, or was only partially successful
   1. What services was that about?
   2. What happened? Why was the initiative not successful or only partially successful?
   3. What could have been done differently?
   4. How can such situations be prevented in the future?
4. How can FHCs be improved? What else can be done so that FHC initiatives are successful in improving services in health facilities?

Is there anything else you would like to add?

Thank you for your contribution.
