# Supplementary material for: Overcoming barriers to access and utilization of maternal, newborn and child health services in northern Nigeria: an evaluation of facility health committees
Source: BMC Health Serv Res. 2018 Feb 9;18:104. doi: 10.1186/s12913-018-2902-7 (PMC5807838; doi:10.1186/s12913-018-2902-7)
Supplement: Supplementary file 3 — In-depth Interview Guide with health providers. (DOCX 29 kb) [file 12913_2018_2902_MOESM3_ESM.docx]

**MCNH2**

**Facility Health Committee Study**

**Provider in-depth interviews**

Provider code

Member gender (circle one) Male / Female

Interviewer code

Name of facility _________________________

Today’s date _______ / _______ / _______

Day Month Year

BACKGROUND

1. How long have you been providing services in this facility?
2. Have you provided services in another facility before this one? If so, how long have you been a health provider overall?
3. What training did you receive before you became a health provider? What additional training have you received since?

FHC MANDATE AND AUTHORITY

1. What do you think is the role of FHCs? What are they meant to accomplish?
2. In your opinion, how much can FHCs influence quality of services?
3. What are the ways the FHCs can help improve quality of services? How else can they contribute to services? What else can they do?
4. When the HFC has a recommendation to make, how do you hear about it? How does it influence your work?
5. Do you feel that FHC members respect the health providers in the facility?

SUCCESS AND FAILURE OF FHC

1. How successful do you think the FHC is in improving services in the health facility?
2. Please tell me about specific instances where your FHC was successful in improving services in the facility? (probe re maternal and child health, if this does not come up spontaneously)
   1. What services were improved?
   2. How where they improved?
   3. Where there any challenges in the process? What were the challenges? How were they overcome?
3. Please tell me about specific instances where your FHC tried to affect change but was not successful, or was only partially successful
   1. What services was that about?
   2. What happened? Why was the initiative not successful or only partially successful?
   3. What could have been done differently?
   4. How can such situations be prevented in the future?
4. How can FHCs be improved? What else can be done so that FHC initiatives are successful in improving services in health facilities?

Is there anything else you would like to add?
