# Supplementary material for: Overcoming barriers to access and utilization of maternal, newborn and child health services in northern Nigeria: an evaluation of facility health committees
Source: BMC Health Serv Res. 2018 Feb 9;18:104. doi: 10.1186/s12913-018-2902-7 (PMC5807838; doi:10.1186/s12913-018-2902-7)
Supplement: Supplementary file 4 — FHC members survey questionnaire. (DOCX 107 kb) [file 12913_2018_2902_MOESM4_ESM.docx]

**MCNH2**

**Facility Health Committee Study**

**FHC member interview**

| **No.** | **Questions** | **Coding categories** |
| --- | --- | --- |
| (a) | State | JIGAWA 1  KANO…………………………………………..…………………………….2  KADUNA 3 |
| (b) | LGA name |  |
| (c) | LGA number | Number |
| (d) | Name of health facility |  |
| (e) | Interviewer code | Code |
| (f) | Date of interview | _______ / _______ / _______  Day Month Year |

| No | Questionnaire and filters | Coding and categories | |
| --- | --- | --- | --- |
|  | **SECTION 1: RESPONDENTS’ BACKGROUND** | | |
| 1 | How old were you on your last birthday? | | Age |
| 2 | Have you attended school? | | Yes………………………………………….………………1  No……………………………………………………….…..2 |
| 3 | What is the highest level of school you attended? | | Quranic……………………………………………….…..1  Primary…………………………………………………2  Secondary………...……..…………………….……..3  Higher……………….……….…………………………4 |
| 4 | What is the highest (class/year) you completed at that level? | | Class/Years at school |
| 5 | Do you do any work for which you earn money? | | Yes 1  No (skip to #7) 2 |
| 6 | What is your **MAIN** occupation, that is, what kind of work do you mainly do? | | Farming 1  Trading 2  Artisan 3  Civil Servant 4  Other ___________________________ 88  (specify) |
| 7 | What is your religion? | | Muslim 1  Catholic 2  Other Christian 3  Other _______________________________ 88  (specify) |
| 8 | How long have you been a member of the FHC committee? | | ­­­Record period in years |
|  | SECTION 2: FHC AND HEALTH SERVICE QUALITY IMPROVEMENT IN HEALTH FACILITIES | | |
|  | FHCs are created in order to improve the quality of of health services in health facilities, now I will ask you questions on your views about the capability of FHCs to contribute to improvement in healthcare services at the health facility | | |
| 9 | Do you think the establishment of FHCs can influence the quality of healthcare services in health facilities? | | Yes…………………………………………………….……1  No……………………………………………..…………2 (Skip to #15) |
|  |  | |  |
| 10 | How much influence can FHCs have on health serve quality improvement? | | Significant influence 1  Limited influence 2  No influence 3 |
| 11 | What areas can FHCs contribute in to improve quality of services at health facilities? | | Health manpower…………………………………………..1  Drugs and commodities supply……………………...2  Equipment and infrastructure………………………..3  Others (Specify) ……………………………………………4 |
| 12 | In what SPECIFIC ways can FHCs contribute to improve quality of services at health facilities? (circle all that apply) | | Increase health provider availability A  Help providers do a better job B  Improve health provider attitudes C  Expend range of services provided D  Increase days or hours of operation E  Increase access of community members to services F  Reduce stock outs of medicines/commodities G  Improve availability of equipment H  Facilitate renovation of the facility I  Enable community mobilization J  Facilitate home services by health workers K  Facilitate outreach services L  Other _______________________________ Z  (specify) |
| 13 | How would you access the level of influence that FHCs can exert to contribute to improve quality of services in health facilities? | | Significant influence 1  Limited influence 2  No influence 3 |
| 14 | Why do you think FHCs can not influence quality of healthcare services in health facilities? | | Facilities don’t listen to recommendations of FHC  FHCs do  Others:……………………………………….. |
|  | **I will like to ask you about the contribution of your FHC to improvement in quality of healthcare at the the health facility** | | |
| 15 | How receptive has facility staff been to recommendations made by your FHC to improve quality of services in the past year? | | Very receptive 1  Somewhat receptive 2  Not receptive 3 |
| 16 | If the FHC makes a recommendation today for an action that will result in improved service quality, do you think facility staff will undertake the action? | | Yes 1  No 2  Maybe 3  I don’t know 8 |
| 17 | How does your FHC engage with the facility when it has a recommendation to improve care? | | Holding meetings with staff of health facility………….1  Communicate recommendations to HMB/SMOH/SPHCDA………………………………………….2  Others (Specify) |
| 18 | Think about recommendations your FHC made in the past year. Were there instances when the FHC positively influenced service provision in the facility? | | Yes 1  No (Skip to 20) …………………………………..……………………..2 |
| 19 | In what ways do you think your FHC activities have influenced the delivery of services in the facility? (circle all that apply) | | Increase health provider availability A  Help providers do a better job B  Improve health provider attitudes C  Expend range of services provided D  Increase days or hours of operation E  Increase access of community members to services F  Reduce stock outs of medicines/commodities G  Improve availability of equipment H  Facilitate renovation of the facility I  Enable community mobilization J  Facilitate home services by health workers K  Facilitate outreach services L  Other _______________________________ Z  (specify) |
| 20 | Think about specific service areas in the facility. Have the FHC improved service quality in the following service areas:  (a) Contraceptive provision  (b) Prenatal services  (c) Delivery of babies  (d) C-sections  (e) Antenatal services  (f) Immunization services  (g) New-born care  (h) Sick child care | | Yes…………………………………………………………………………..1  No…………………………………………………………………………….2 |
| 21 | Overall, do you think the committee has been successful in carrying out its responsibilities in improving quality of service delivery at the facility? | | Yes 1  No ………………………………………………………………………………2 |
| 22 | How successful do you think your committee has been in influencing the quality of services in the facility since you joined the FHC? | | Very successful 1  Somewhat successful 2  Not successful(skip to 24)l 3 |
| 23 | What do you think are factors that are responsible for the success of the committee in contributing effectively to quality of care? (circle all that apply) | | Support of the community A  Support of facility staff B  Commitment of FHC members C  Commitment of facility staff D  Adequate funding E  Appropriate training F  Other _______________________________ Z  (specify) |
| 24 | Can you think of factors that reduce the effectiveness of FHC contributing, or prevent the FHC from contributing to improved service delivery in the facility? (circle all that apply) | | Misconceptions among community members A  Misconceptions among facility staff B  Lack of commitment of FHC members C  Lack of support from community members D  Opposition of facility staff E  Inadequate funding F  Insufficient training G  Other _______________________________ Z  (specify) |
| 25 | What are challenges that FHC members face that reduce the effectiveness of their contribution to quality of care at the facility? (circle all that apply) | | Misconceptions among community members A  Misconceptions among facility staff B  Lack of commitment of other FHC members C  Lack of support from community members D  Opposition of facility staff E  Inadequate funding F  Insufficient training G  Other _______________________________ Z  (specify) |
| 26 | Do you think that the FHC receives enough support from community members? | | Yes 1  No. 2 |

As I told you earlier, this survey is administered to several hundred FHC members in three MNCH2 states in northern Nigeria. After we look at initial result, we will select some of the respondents and invite them to participate in focus groups, to get a more in-depth understanding of our findings. If you are selected to participate we will contact you, explain the study in more detail, and ask for your consent to participate.

May I have your permission to contact you if you are selected?

Yes 1

No 2
